# Locking suture repair versus ligament augmentation—a biomechanical study regarding the treatment of acute lateral collateral ligament injuries of the elbow

**DOI:** 10.1007/s00402-022-04337-0

**Published:** 2022-01-22

**Authors:** Nadine Ott, Arne Harland, Fabian Lanzerath, Tim Leschinger, Michael Hackl, Kilian Wegmann, Lars Peter Müller

**Affiliations:** Department of Trauma and Orthopedic Surgery, University Hospital Cologne and Faculty of Medicine, University of Cologne, Kerpener Street 62, 50937 Cologne, Germany

**Keywords:** Posterolateral rotatory instability, LCL tear, LCL repair, Ligament augmentation, Stability, Cyclic loading

## Abstract

**Background:**

Lateral collateral ligament (LCL) tears are frequently observed in fractures and dislocations of the elbow. Recent biomechanical evidence suggests that additional ligament augmentation may improve repair stability. The aim of this biomechanical in-vitro study was to compare the resistance of a locking suture repair of the LCL with a ligament augmentation technique.

**Material and methods:**

Eight fresh frozen cadaveric elbows were evaluated for stability against varus/posterolateral rotatory forces (3 Nm). A strain gauge (µm/m; negative values) was placed at the origin and insertion of the lateral ulnar collateral ligament (LUCL) and cyclic loading was performed for 1000 cycles. We analyzed three distinct scenarios: (A) native LCL, (B) locking transosseou suture repair of the LCL, (C) simple LCL repair with additional ligament augmentation of the LUCL.

**Results:**

The mean measured strain was − 416.1 µm/m (A), − 618 µm/m (B) and − 288.5 µm/m (C) with the elbow flexion at 90°; the strain was significantly higher in scenario B compared to C (*p* = .01). During the cyclic load (1000) the mean measured strain was − 523.1 µm/m (B) and − 226.3 µm/m (C) with the elbow flexion at 60°; the strain was significantly higher in scenario B compared to C (*p* = .01). No significant difference between the first and the last cycles was observed (*p* = .09; *p* = .07). One failure of the LCL repair was observed after 1000 cycles; none of the ligament augmentations failed.

**Conclusion:**

Ligament augmentation (C) provides higher resistance compared to the native LCL (A) and to the locking suture repair technique (B). Both techniques, however, hold up during 1000 cycles. While ligament augmentation might enhance the primary stability of the repair, future clinical studies have to show whether this increase in resistance leads to negative effects like higher rates of posttraumatic elbow stiffness.

**Level of evidence:**

Basic science study, biomechanics.

## Introduction

Lateral collateral ligament (LCL) injuries are frequent elbow pathologies in elbow trauma. The elbow is the second most dislocated joint of the upper extremity after the shoulder [1]. In case of ligamentous dislocations, LCL injuries are most frequently accompanied lesions. Simple ligamentous lesions can be treated conservatively [2]. However, there is a subset of patients who experience recurrent instability or subluxation; a posterolateral rotatory instability (PLRI) could result [3–5]. PLRI of the elbow is associated with an insufficient lateral collateral ligament complex (LCL) [6–9]. When surgical treatment is performed, LCL repair is crucial to avoid persisting posterolateral rotatory instability. Recently, ligament augmentation techniques have gained popularity [2, 9–19].

Besides primary repair and autograft or allograft reconstructions, the use of ligament brace techniques has been studied and reported to be stronger biomechanically [1, 10, 13–15, 19–21]. The goal of all these procedures is an anatomic reconstruction of the LCL for restoration of functional elbow stability and ligament repair with a locking transosseous suture is still preferred by many surgeons [22].

However, to the Authors knowledge, there have been no biomechanical in-vitro study that evaluated the use of additional ligament augmentation and this LCL repair according to the locking transosseous suture technique [22] compared to the native LCL. Therefore, the present study aimed to analyze the resistance of primary repair, additional ligament augmentation of LCL and the native LCL after 1000 cycles rotational load.

## Material and methods

### Specimen preparation

For this biomechanical study, nine fresh frozen cadaveric elbows from 3 male and 6 female donors were available. The mean age at the time of death was 73 (min. 65, max. 91, SD 12 years). The specimens were stored at − 20 °C and thawed at room temperature 12–14 h before dissection and biomechanical testing. Fluoroscopic and clinical examinations were performed to exclude specimens with osteoarthritis or signs of previous surgery and trauma. The soft tissue of the proximal humerus and the forearm was preserved.

**Fig. 1:**
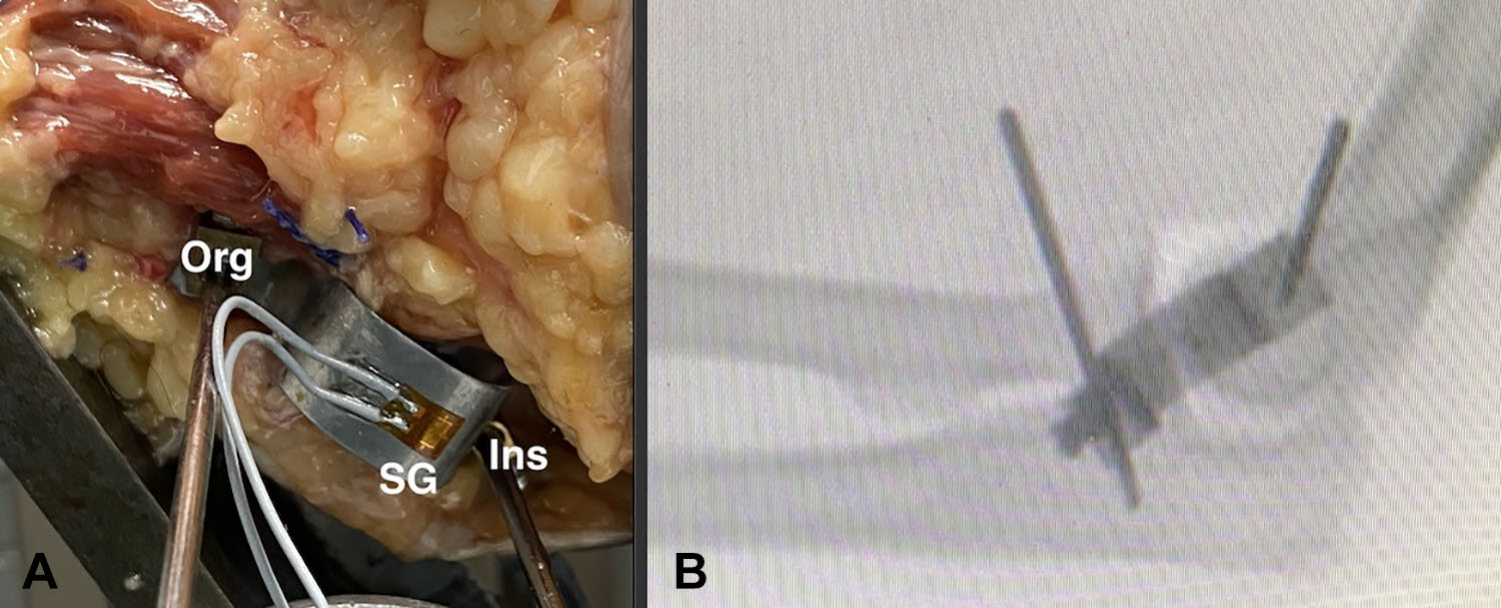
The titanium omega augmented with a strain gauge, SG, (4-wire 120 Ω, Vishay Inc., Malvern, PA, USA) was fixed at the origin (Org) and insertion (Ins) of the LUCL (**A**). A posterolateral rotatory instability with ulno-humeral displacement resulted in a deformation of the fixed omega. By fluoroscopy the anatomical position was verified (**B**); in each scenario closure of the fascia overlying the Kocher interval was performed (PDS 0, Ethicon Inc., Bridgewater, NJ, USA)

Strain of the LUCL was measured indirectly via strain gauges. A strain gauge (4-wire; 350 Ohm; Vishay Inc., Malvern, PA, USA) was fixed on top of a custom-made sensor (Steel, Dx 51) with M-Bond (Vishay Inc., Malvern, PA, USA); the sensor takes the form of a omega. The form of the omega allows the efficient transfer of the applied force through the strain gauge (Fig. 1A). While the measurement the deformation of the sensor was transferred through the strain gauge. A calibration of each strain gauge was performed. Axial deformation was transferred through the strain gauges and therewith resulted in a deflection of the measuring device, which was digitally documented via a software in µm/m (MGCplus Fa. HBM, Darmstadt, Germany). To reduce measurement errors, a 4-wire strain gauge was used.

The sensor augmented with a strain gauge (4-wire 120 Ω, Vishay Inc., Malvern, PA, USA) was fixed along the course of the LUCL with the help of threaded k-wires placed at the origin and insertion of the LUCL (Fig. 1B). Posterolateral rotation of the forearm resulted in a deformation of the sensor and the sensor transferred the applied force through the strain gauge.

### Scenario A

In scenario A, the ligaments and the fascia of the forearm remained intact. After testing the specimens in the intact state, a lateral Kocher approach was performed and the LCL along with the common extensor origin were sharply detached from the distal humerus.

### Scenario B

**Fig. 2:**
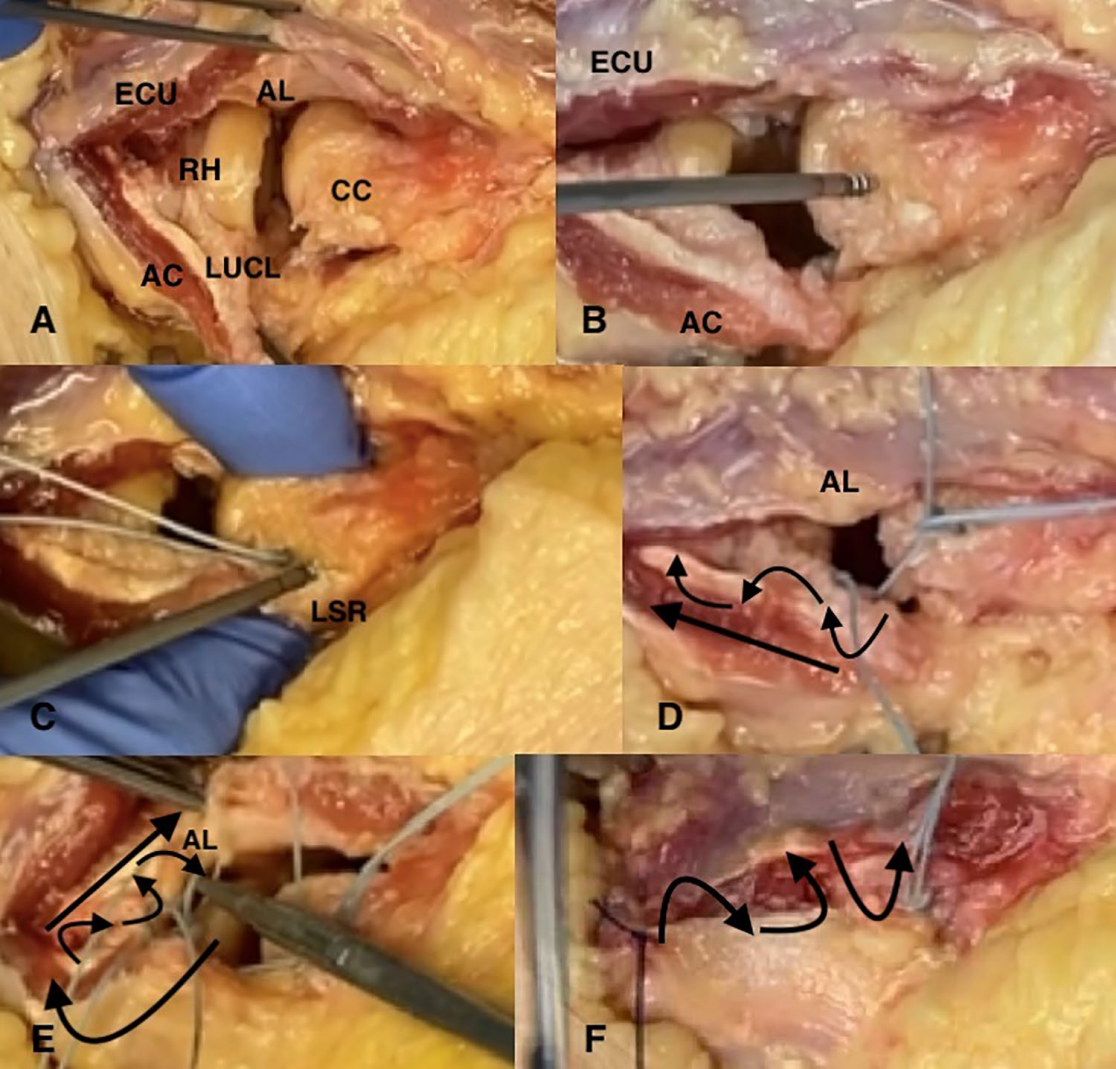
Lateral Kocher approach was performed; extensor carpi ulnaris (ECU), aconeus muscle (AC), anular ligament (AL); humeral isometric point was located by identifying the intersection point in which the lateral condyle is bisected by a line from the center of the radial head (RH) both in 90° of flexion and extension (**A**). In contrast to the technique described in Green’s chapter [22], we have used two 2.4 mm FASTak (Fa. Arthrex). The first was placed at the axis of motion (the center of the arc of curvature of the capitulum; CC) (**B**) and the second placed posterior to the lateral supracondylar ridge; LSR (**C**). Suture passers are placed to facilitate the repair. A locking suture technique is employed to gain a secure hold of LUCL (**D**). The interval between radial collateral and LUCL and the annular ligament (AL) are closed at this suture is brought back to the lateral epicondyle (**E**). The ligament sutures are pulled into the FASTak in the distal humerus with maintaining the forearm in pronation and avoiding varus forces while tying the sutures (**F**)

In scenario B, a locking suture repair of the LCL was performed, modified according to the technique published in Green’s chapter [22]: first, two suture anchors (FASTak, 2.4 mm, Arthrex Inc., Naples, FL, USA) were placed in the center of rotation of the capitulum and in the lateral supracondylar ridge. A locking suture was then placed in the LUCL from proximal to distal over the course of 3 cm with one suture limb of the suture anchor placed in the center of rotation. On the way back, the radial collateral ligament and the annular ligament were incorporated in the repair to close the interval between the RCL and the LUCL. A sliding knot was performed to secure the LCL back to its origin while holding the elbow in 90° of flexion and in full pronation. The second suture anchor was used for refixation of the common extensor origin with a mattress suture (Fig. 2). Closure of the fascia overlying the Kocher interval was performed (PDS 0, Ethicon Inc., Bridgewater, NJ, USA).

### Scenario C

**Fig. 3:**
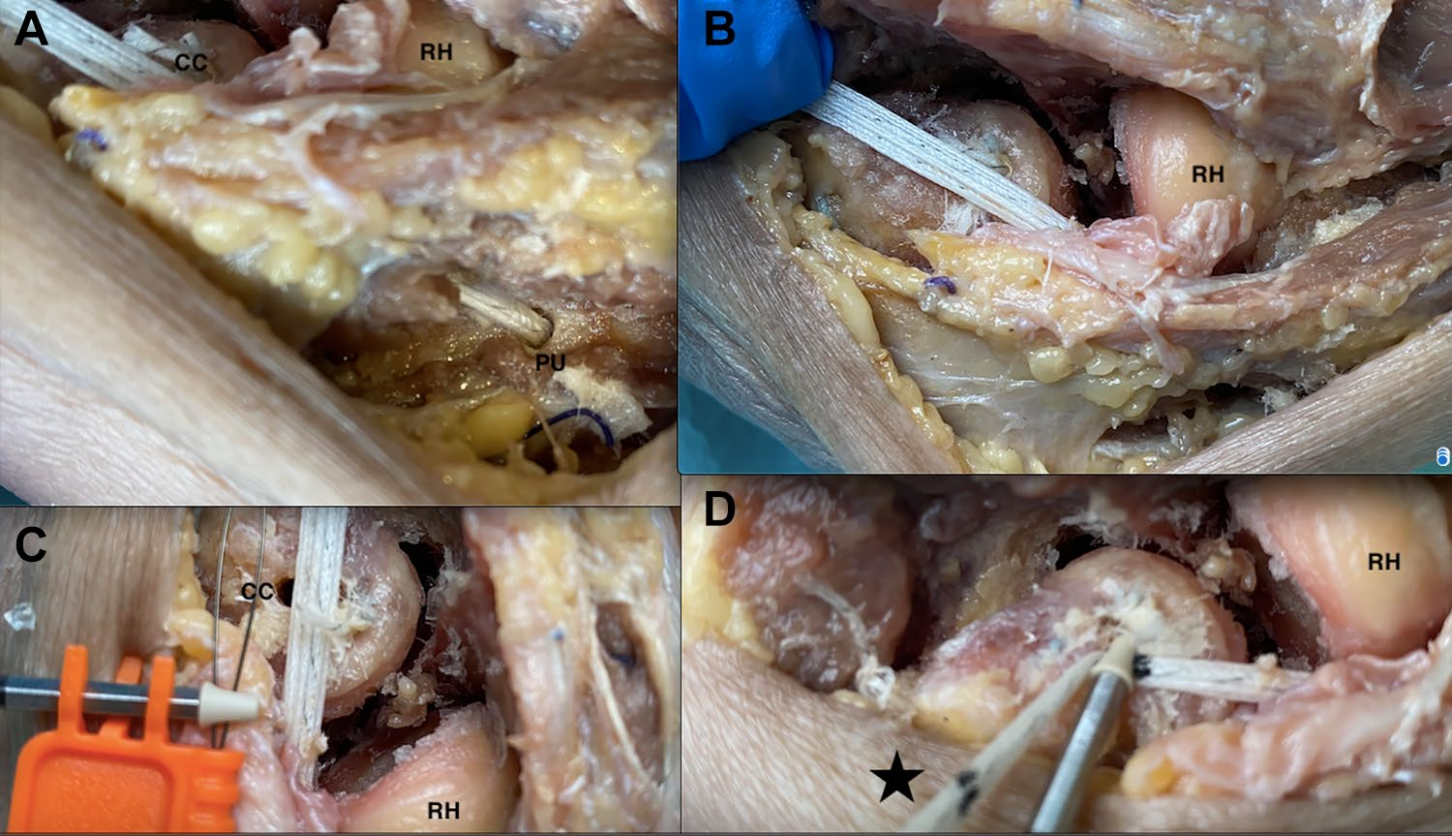
two drill holes were employed: one in the capitulum at the center of rotation (CC); and another in the proximal ulna (PU) directly posterior to the supinator crest and approximately 1 cm distal to the articular surface of the radial head (RH) (**A**). A suture anchor loaded with a suture tape (3.5 mm PEEK SwiveLock anchor; FiberTape^®^, Arthrex Inc., Naples, FL, USA) was inserted in the distal drill hole. The lateral capsule was closed with interrupted 2–0 sutures. The suture tape was then spanned over the lateral capsule (**B**). The entry point of the tape into the humeral bone tunnel was marked with a pen. In the next step, fixation of the tape was performed by inserting a second suture anchor into the distal humerus with respect to the pen marking (black star) in order not to over-tighten the augmentation (**C**) The remaining suture ends of the suture tape were used to repair the LCL and the common extensor origin back to bone with a simple suture. The tape was then spanned over the LUCL with elbow at 30° flexion and a pronated forearm. To control the tight, the sutures were marked at the laser line (**D**)

In scenario C, two drill holes were employed: one in the capitulum at the center of rotation; and another in the proximal ulna directly posterior to the supinator crest and approximately 1 cm distal to the articular surface of the radial head. A suture anchor loaded with a suture tape (3.5 mm PEEK SwiveLock anchor; FiberTape^®^, Arthrex Inc., Naples, FL, USA) was inserted in the distal drill hole. The lateral capsule was closed with interrupted 2–0 sutures. The suture tape was then spanned over the lateral capsule. The entry point of the tape into the humeral bone tunnel was marked with a pen. In the next step, fixation of the tape was performed by inserting a second suture anchor into the distal humerus with respect to the pen marking in order not to over-tighten the augmentation. The remaining suture ends of the suture tape were used to repair the LCL and the common extensor origin back to bone with a simple suture. The tape was then spanned over the LUCL with elbow at 30° flexion and a pronated forearm. To control the tight, the sutures were marked at the laser line. The anchor was placed at the marked sutures and fixed at the proximal drill hole (Fig. 3). Identically to scenario B, the fascia was closed.

To keep the measurements errors low, a 4-wire strain gauge was used.

### Biomechanical testing set-up

**Fig. 4:**
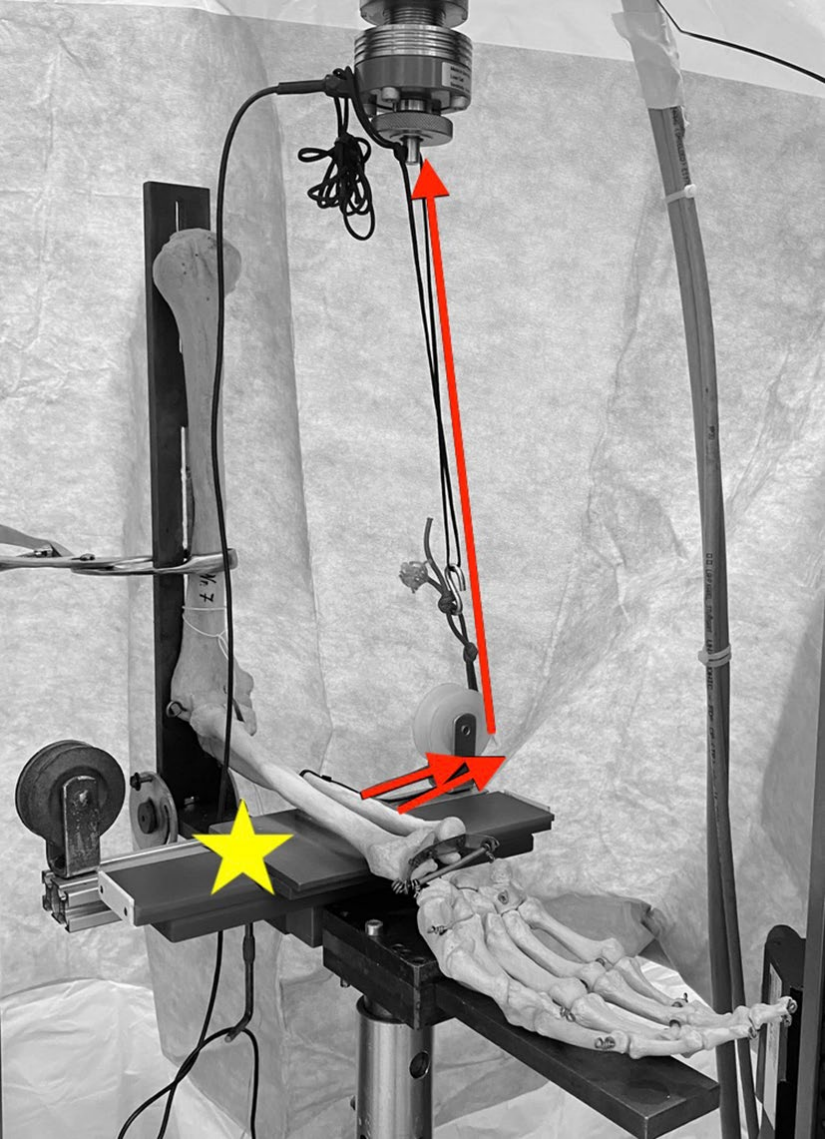
upward movement of the mobile traverse (red arrows) resulted in posterolateral rotatory/varus force, depending on wire deflection (yellow star). This biomechanical testing set-up has been used by previous study [8, 9, 23]

The humeral shaft was secured to a custom-made testing fixture with two mounting clamps. The hinged testing fixture allowed for movement of the elbow joint from 60° to 90° and was mounted onto a servohydraulic universal testing machine (ZwickRoell; Ulm, Germany). A mounting bolt was securely fixed to the lateral side of the ulnar shaft 10 cm distal to the center of rotation. A synthetic wire connected the bolt to the mobile traverse of the testing machine. Reels were used for deflection of the wire. Thereby, upward movement of the mobile traverse resulted in posterolateral rotatory/varus force, depending on wire deflection (Fig. 4). This biomechanical testing set-up was used in previous studies [8, 9, 23]. Stability in each scenario was evaluated with the elbow in 60° and 90° of flexion.

A maximal rotational moment of 3 Nm at 0.5 Hz was applied. Each scenario was performed with elbow flexion at 60° and 90° with 10 rotational cycles. In scenario B and C, the strain was measured with elbow flexion at 60° through 1000 rotational cycles. The resulting deformation of the sensor was transferred through the strain gauge (µm/m). This testing method was designed to simulate the immediate postoperative period when failure is most likely to occur by repetitive movements. By performing temporary arthrodesis of the distal radio- ulnar joint with two 2.0-mm Kirschner wires, measurements could be obtained in supination. The specimen remained in the identically biomechanical testing set-up for each scenario. Thereby, measurement errors between each scenario were kept low. The repair techniques were performed by the senior author (L.P.M.).

In our biomechanical testing set-up, negative values of the strain gauge resulted in a deformation of the used omega.

### Statistical analysis

The data collected were analyzed using the SPSS statistical program. Normal distribution was tested by Kolmogorov–Smirnov. A *t* test and ANOVA-test was performed to detect any statistically significant differences. We used descriptive statistics to summarize the means and standard deviations. The level of significance was defined as a *p* value of < 0.05.

### Ethical considerations

The local ethics committee approved this work and informed consent was obtained from each volunteer included in this study (Ethical Committee of the Medical Faculty of the University of Cologne—VT (No: 20-1369). This study followed the guidelines for experimental investigation with human subject required by our institution.

## Results

**Fig. 5:**
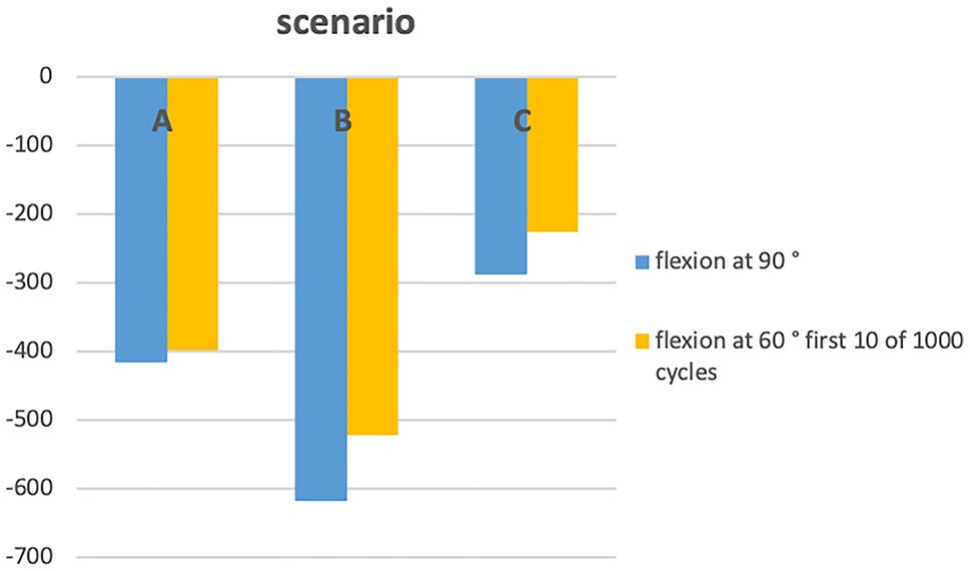
overview of the results, y-axis presents the measured strain (µm/m) in scenario A (native), B (repair) and C (ligament augmentation) (x-axis)

Table 1 and Fig. 5 summarized the main results.

**Table 1:** summarized the main results; measured strain in µm/m in scenario A–C and the position of the elbow with *p* values

	Scenario			*p* value		
	A (strain in µm/m)	B (strain in µm/m)	C (strain in µm/m)	A/B	B/C	A/C
Position of the elbow						
Flexion at 90°	− 416.1	− 618	− 288	.03	.07	.34
Flexion at 60°						
First 10 of 1000	− 399	− 523.1	− 226.3	.07	.01	.08
Last 10 of 1000		− 404.2	− 278		.12

The mean measured strain was − 416.1 µm/m (A), − 618 µm/m (B) and − 288.5 µm/m (C) with the elbow flexion at 90°; the strain was significantly higher in scenario B compared to C (*p* = 0.01). During the cyclic load the mean measured strain was − 523.1 µm/m (B) and − 226.3 µm/m (C) with the elbow flexion at 60°; the strain was significantly higher in scenario B compared to C (*p* = 0.01). No significant difference between the first and the last cycles were observed (*p* = 0.09; *p* = 0.07). In scenario B, one failure after 1000 cycles was observed. The suture was closed, the ligament sutures which were pulled back to the FASTak lost their tightness.

In scenario C, no failure of the ligament augmentation was detected.

### 90° of elbow flexion

In scenario A, the mean measured strain was − 416.1 µm/m (min. 84 µm/m; max. − 950 µm/m, SD 315.4 µm/m). In scenario B, the mean measured strain was − 618 µm/m (min. 177 µm/m; max. − 1065 µm/m, SD 325.6 µm/m) and in scenario C, the mean measured strain was − 288.5 µm/m (min. − 11 µm/m; max. − 740 µm/m, SD 243.9 µm/m).

### 60° of elbow flexion

In scenario A, the mean measured strain was − 399 µm/m (min. − 100 µm/m; max. − 770 µm/m; SD 219 µm/m) during 10 cycles. In scenario B, in the first cycles of 1000 cycles the strain was -523.1 µm/m (min. − 170 µm/m; max. − 660 µm/m; SD 149.6 µm/m) and in the last cycles it was − 404.3 µm/m (min. − 85 µm/m; max. − 760 µm/m, SD 232.9 µm/m). In scenario C, in the first cycles of 1000 cycles the strain was − 226.3 µm/m (min. − 6 µm/m; max. − 680 µm/m; SD 244.8 µm/m) and in the last cycles it was − 278 µm/m (min. − 65 µm/m; max. − 650 µm/m, SD 217.5 µm/m).

## Discussion

The most important finding of our study is, that ligament augmentation of the LUCL shows significant less displacement compared to the native LCL and the locking suture repair technique [22]. After 1000 cycles no failures in the ligament augmentation group was observed. The ulno-humeral displacement by the deformation of the omega was not increased significantly during the 1000 loading cycles. In the repair group, macroscopic loosening of the locking suture was observed in one case following cyclic loading.

As mentioned previously, Melbourne et al. [19] showed that suture tape augmentation of the LUCL is associated with significantly higher load to failure than repair or reconstruction alone. They described a protective effect of the ligament augmentation on the underlying repaired ligament. This can be confirmed by the results of the present study.

Recently, Ellwein et al. [14] observed a higher load to failure after LUCL repair with additional ligament bracing than repair alone. In the present study, a load to failure was not performed. We used the strain gauge to evaluate the ulno-humeral displacement by the deformation of the omega continuously during 1000 cycles. Comparing the measured strain at the first 10 and the last 10 of 1000 cycles, no significant difference could be observed in the internal brace and the repair group.

Greiner et al. [21] published the clinical results after LUCL repair augmented with ligament bracing. Seventeen patients with acute or subacute posterolateral elbow instability as a result of dislocation or fracture dislocation were treated with open LUCL refixation and non-absorbable suture tape augmentation. The elbows were actively mobilized immediately after the operation and a maximum bracing period of 3 days. All patients were without recurrent instability at the time of follow-up. Despite rehab, range of motion was not very good at ten months. Patients in this study had a mean extension lag of 10°. While the re-operation rate was low. Their postoperative time to full mobilization was significantly lower than of other LUCL studies. Taking their observations, LUCL repair with ligament augmentation might shorten rehabilitation based on a resistance. However, Fraser et al. have shown that LCL repair using transosseous sutures is a useful technique to restore initial elbow kinematics [24].

Future research will need to clarify whether ligament augmentation leads to higher rates of postoperative elbow stiffness due to the augmentation, however, the LCL repair as well as the ligament augmentation hold up during 1000 cycles. Although, an increased displacement after 1000 cycle was measured in the ligament augmentation group. Our results have shown greater resistance in the ligament augmentation group.

Compared to the native LCL, the resistance of the ligament augmentation might be higher. So, it remains unknown whether higher resistance due to the increased rigidity of the repair might result in higher rates of postoperative elbow stiffness. Consequently, it may be not useful to perform additional ligament augmentation in every case of elbow instability. In our clinical experience, the decision for repair alone or additional augmentation was based on the severity of instability and the accompanied lesions.

The present study has several limitations. Firstly, the small sample size may pose the risk of a type two error. Due to the difficulty of procuring suitable cadaveric specimens, this problem is commonly encountered in biomechanical research. Another inherent weakness of cadaveric biomechanical studies is the age of the specimens relative to the patient population of interest. Finally, the study conditions do not accurately recreate the physiological reality that the studies portend to examine. However, to our knowledge, this is the first study which has remained all the soft tissues. The last drawback of the present study the cyclic loading (1000) just being performed in a single elbow position and single degree of freedom.

## Conclusion

Ligament augmentation provides higher resistance compared to the native LCL and to the locking suture repair technique. Both techniques, however, hold up during 1000 cycles. While ligament augmentation might enhance the primary stability of the repair, future clinical studies have to show whether this increase in resistance leads to negative effects like higher rates of posttraumatic elbow stiffness.
